# Plating Silver Bases on Which Artificial Teeth Are Mounted with Gold

**Published:** 1857-07

**Authors:** 


*Plating Silver Bases on which Artificial Teeth are Mounted with Gold.*—Dr. A. E. Pursell, dentist, of Madison, Indiana, has recently shown to the senior editor some pieces of silver plate so thickly covered with gold, that the amount of friction to which artificial dentures are exposed, would not remove it for years. By the process which Dr. P. employs, one or more leaves of No. 6, 8 or 10 gold foil are placed upon and united directly to the silver plate, and so perfectly that the piece may be afterwards stoned and burnished or polished, giving to it as beautiful a finish as would be made if the entire plate were gold. In the application of temporary sets of teeth mounted on silver, and in cases where the patient is not able to incur the expense of gold, this process of plating, which is very simple and may be done by any one, can be advantageously employed. It is, also, equally valuable for covering those parts of a gold base on which solder has run, thus preventing any unpleasant copperish or galvanic taste.

Dr. P. has promised to furnish in a short time, a full description of his method of plating with gold. We hope to get it in time for the next number of the Journal.

